# Psychological Need Satisfaction, Self-Determined Motivation, Burnout, and Dropout Intention in Korean Water Polo Athletes: A Cross-Sectional Structural Equation Model

**DOI:** 10.3390/healthcare14213748

**Published:** 2026-11-03

**Authors:** Seungjae Lee, Buom Kim, Byoungwook Ahn

**Affiliations:** 1 Department of Sports Leisure, Nambu University, Gwangju 62271, Republic of Korea; 2 Department of Physical Education, Metropol Campus, Kyungdong University, Yangju-si 11458, Republic of Korea; 3 Department of Leisure Marine Sports, Seosan Campus, Hanseo University, Seosan-si 31692, Republic of Korea

**Keywords:** basic psychological needs, self-determined motivation, athlete burnout, dropout intention, water polo, Self-Determination Theory

## Abstract

**Highlights:**

**What are the main findings?**

- In a cross-sectional sample of 173 Korean water polo athletes, athlete burnout was strongly associated with dropout intention, although the two constructs overlapped substantially and their discriminant validity was not conclusively established.
- Need satisfaction was indirectly associated with dropout intention via burnout alone and sequentially via self-determined motivation and burnout; this sequential association depended on the motivation index and reflected mainly amotivation rather than autonomous motivation.

**What are the implications of the main findings?**

- Monitoring burnout-related experiences, particularly sport devaluation and amotivation, may be a theory-informed consideration for coaches and practitioners in small, structurally constrained sports such as water polo.
- Whether need-supportive coaching reduces burnout or dropout was not tested in this study and requires longitudinal and intervention research.

**Abstract:**

**Background/Objectives:** Grounded in Self-Determination Theory, this cross-sectional study examined associations among basic psychological need satisfaction, self-determined motivation, athlete burnout, and dropout intention in Korean water polo athletes. **Methods:** High-school, collegiate, and general-level players (*n* = 173; 162 male) completed online questionnaires; the outcome was dropout intention, not observed dropout. Structural equation modeling with bias-corrected bootstrapping and sensitivity analyses was conducted. **Results:** Need satisfaction was positively associated with self-determined motivation, both were negatively associated with burnout, and burnout was strongly associated with dropout intention (β = 0.794), whereas direct paths to it were not significant. Need satisfaction was indirectly associated with dropout intention via burnout alone and sequentially via motivation and burnout (B = −0.313, 95% BC CI [−0.667, −0.123]). Burnout and dropout intention overlapped substantially (latent r = 0.852; Fornell–Larcker criterion not met), although the association remained strong without sport devaluation (β = 0.663). The sequential association reflected mainly amotivation and was not significant with a latent autonomous-motivation factor. **Conclusions:** Burnout-related experiences, particularly sport devaluation, were strongly associated with dropout intention, but discriminant validity was not conclusively established. The findings highlight amotivation rather than a general protective role of autonomous motivation; longitudinal research is needed.

## 1. Introduction

Water polo is a physically and psychologically demanding team sport that combines sustained swimming, explosive movement, ball handling, tactical decision-making, and frequent physical contact. Because training and competition involve repeated high-intensity activity with limited recovery, the sport imposes substantial physiological demands and produces accumulated fatigue [1,2,3]. Athletes must in addition adapt to shifting tactical roles, to performance evaluation, to interpersonal coordination, and to competition for playing opportunities. Sustained participation therefore calls not only for physical conditioning and technical competence but also for the psychological resources that help athletes to sustain motivation and to cope with prolonged training and competitive pressure.

These demands may be particularly consequential in Korean water polo, which is sustained by a limited number of teams and a tournament-centred competition structure, and in which training infrastructure, athlete-development pathways, and competitive opportunities have been described as restricted [4]. The women’s national programme, for example, was first assembled in 2019 and was not established as a permanent squad until 2021 [5,6,7], and a long-term analysis of the Korean National Sports Festival has shown that competitive strength has become increasingly concentrated among a small number of teams, particularly in the adult division [8]. In such a small system, the withdrawal of even a few athletes may affect individual teams, which makes athlete dropout a relevant concern for the sport.

International evidence likewise indicates that motivational processes are relevant to continued participation in water polo. Research with youth water polo players has shown that coaches’ motivating style is related to players’ motivation and sport commitment [9]. Attrition may be especially consequential in Korea, where the active player and team base is small [4,8]. Dropout intention does not necessarily lead to actual withdrawal, but it is a relevant proximal indicator of possible future dropout and may help to identify athletes who are considering leaving the sport.

Self-Determination Theory (SDT) offers a useful framework for explaining these psychological processes. SDT posits three basic psychological needs—autonomy, competence, and relatedness [10,11]. Autonomy denotes a sense of volition and personal choice, competence the feeling of being effective and capable, and relatedness the feeling of being accepted and meaningfully connected to others. Where these needs are met, individuals are more likely to internalize their participation and to engage for relatively autonomous reasons; where they are not, both motivational quality and psychological functioning may deteriorate. The present study focuses on need satisfaction; need frustration, the active thwarting of these needs, is a conceptually distinct experience that was not assessed, and low need satisfaction should therefore not be equated with need frustration.

In competitive sport, need satisfaction is shaped by structured training, by performance expectations, by coaching practice, and by relationships with teammates. Athletes who experience volition, competence, and supportive relationships are more likely to develop self-determined forms of motivation, that is, to participate on the basis of intrinsic interest, personal value, and integrated goals rather than of external pressure, guilt, or an absence of motivation. Previous sport research has consistently linked the satisfaction of autonomy, competence, and relatedness with more autonomous forms of motivation [10,12]. Need satisfaction may therefore provide an important psychological foundation for sustaining intensive training and competitive participation.

Motivational quality may also bear on vulnerability to athlete burnout, a multidimensional syndrome marked by emotional and physical exhaustion, a reduced sense of accomplishment, and sport devaluation [13,14]. Burnout may arise where prolonged sport demands meet insufficient psychological resources, limited control, declining achievement, or diminished meaning. Athletes with more self-determined motivation may be less vulnerable, since they are more likely to find training and competition personally meaningful, whereas controlled or amotivated participation may render sport obligatory and psychologically exhausting.

Empirical findings support these relationships. Basic psychological need satisfaction has been negatively associated with athlete burnout across competitive sport settings, and meta-analytic evidence indicates that athletes reporting greater need satisfaction generally experience less exhaustion, a less reduced sense of accomplishment, and less sport devaluation [15]. De Francisco et al. [12] further showed that need satisfaction was related to both engagement and burnout, and that these relationships were partly explained by self-determined motivation. Need satisfaction may therefore be associated with lower burnout both directly and indirectly, through higher-quality motivation.

Burnout, in turn, may be closely associated with the intention of athletes to discontinue participation. Athletes experiencing persistent exhaustion, a diminished sense of accomplishment, and devaluation may come increasingly to question whether the benefits of continued participation outweigh its psychological and physical costs. From a sport commitment perspective, withdrawal becomes more likely as enjoyment and perceived rewards decline while costs and stress rise [13,16]. Because the present study did not measure core sport-commitment variables such as enjoyment, involvement alternatives, personal investments, or social constraints, this perspective is used as a complementary interpretive lens rather than as a directly tested framework. Burnout may therefore be closely associated with dropout intention among athletes who are still actively participating.

Self-determined motivation and basic psychological need satisfaction may also show direct associations with dropout intention. Athletes who participate because they value, enjoy, and personally endorse their involvement are generally more likely to persist than those whose participation is driven by pressure or obligation, and a meta-analysis of prospective studies found that motivation-related constructs were among the strongest predictors of dropout from adolescent team sport [17]; need-related experiences have also been linked to dropout intentions through motivation [18]. Both motivational quality and need satisfaction may accordingly retain direct negative associations with dropout intention even once burnout has been taken into account.

Taken together, SDT provides a theoretical basis for a hypothesized ordering in which greater psychological need satisfaction is associated with more self-determined motivation, greater self-determined motivation with lower athlete burnout, and lower burnout with lower dropout intention. This pattern may be particularly relevant in water polo, where athletes face substantial physical demands, intensive team interaction, competitive pressure, and limited career pathways. Few studies, however, have examined need satisfaction, self-determined motivation, athlete burnout, and dropout intention simultaneously within a single structural model. Existing water polo research on motivation has concentrated on the interpersonal behaviour of coaches and on separate bright and dark motivational pathways [9], rather than on an integrated pattern linking the need experiences of athletes to their withdrawal intentions.

This gap is particularly evident in Korea, where water polo research has concentrated on physical fitness and anthropometric characteristics [19,20], on training and performance [21], and on technical or tactical game characteristics [22,23]. Psychological research remains limited and has addressed mainly performance-oriented mental skills rather than the motivational processes related to continued participation [24]. Given the small competitive base, identifying the psychological factors associated with dropout intention carries both theoretical and practical importance for coaches, sport organizations, and athlete-support personnel.

**Figure 1:**
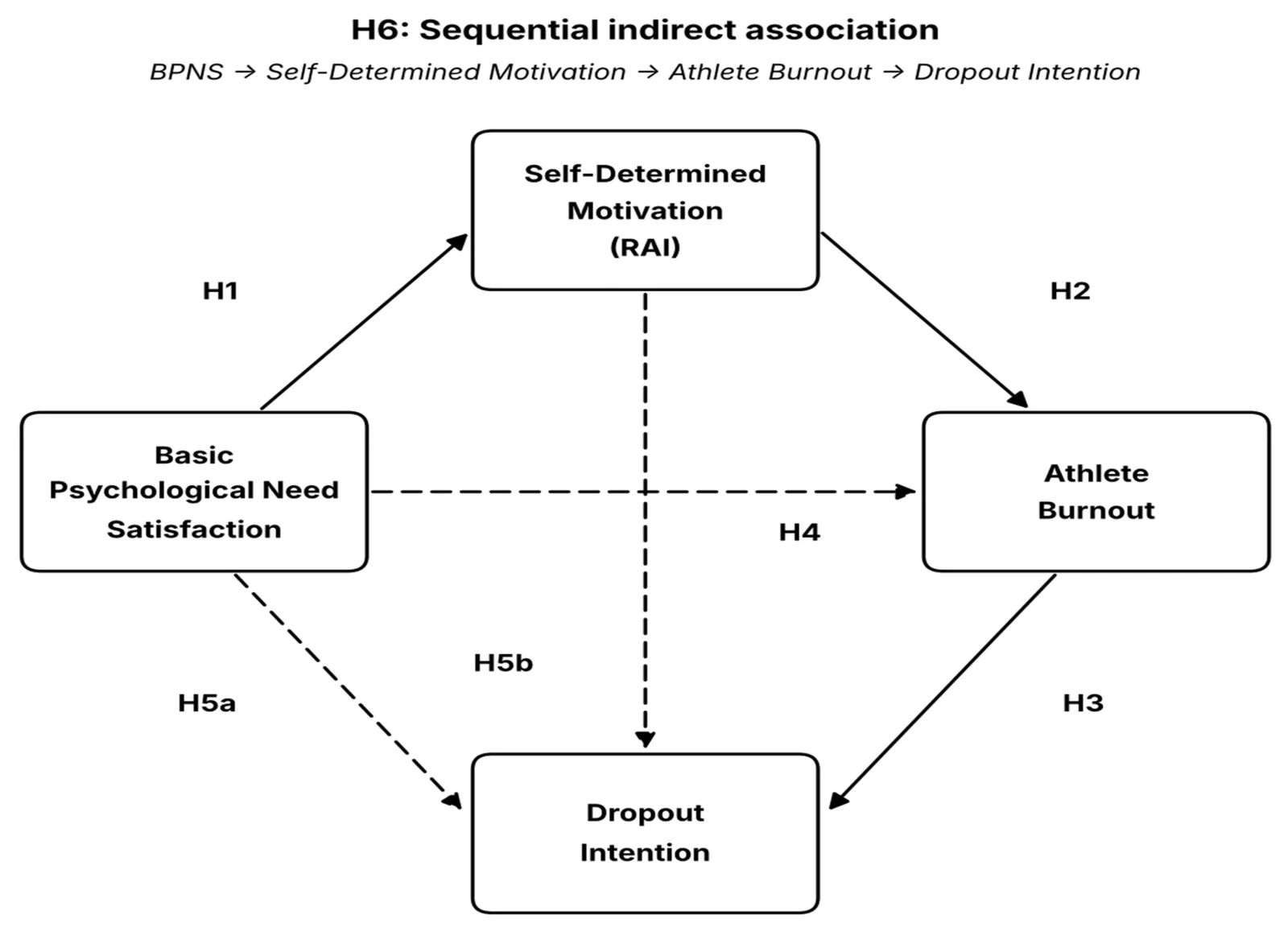
Hypothesized structural model of the associations among basic psychological need satisfaction, self-determined motivation, athlete burnout, and dropout intention in Korean water polo athletes. Solid arrows denote the hypothesized sequential paths (H1–H3), which together define the sequential indirect association tested in H6, and dashed arrows denote the additional hypothesized direct paths (H4–H5). The ordering of the variables is theoretically specified and was not established empirically.

This study accordingly examined, using a cross-sectional design, the associations among basic psychological need satisfaction, self-determined motivation, athlete burnout, and dropout intention in currently active Korean water polo athletes, including adult club and masters players at the general level. Drawing primarily on SDT, it tested whether the data were consistent with a theoretically specified ordering in which need satisfaction is positively associated with self-determined motivation, self-determined motivation is negatively associated with burnout, and burnout is positively associated with dropout intention. It further examined whether need satisfaction and self-determined motivation retained direct associations with dropout intention once burnout was included in the model. Because all variables were measured at one time point, the hypotheses concern statistical associations in the hypothesized direction rather than causal effects. The hypothesized model is presented in Figure 1, and the following hypotheses were proposed: H1. Basic psychological need satisfaction is positively associated with self-determined motivation. H2. Self-determined motivation is negatively associated with athlete burnout. H3. Athlete burnout is positively associated with dropout intention. H4. Basic psychological need satisfaction is negatively associated with athlete burnout, controlling for self-determined motivation. H5. Basic psychological need satisfaction (H5a) and self-determined motivation (H5b) are each negatively associated with dropout intention, controlling for burnout. H6. Basic psychological need satisfaction shows a significant sequential indirect association with dropout intention via self-determined motivation and athlete burnout within the specified model.

## 2. Materials and Methods

### 2.1. Participants

Participants were water polo players who were actively taking part in high school, collegiate, or general-level teams in South Korea at the time of data collection. The general level included adult club (recreational) and masters players as well as elite adult athletes. Because the player population is small, recruitment was conducted broadly across the national water polo community.

To be eligible, participants had to (a) be currently active water polo players affiliated with a high school, collegiate, or general-level team (including adult club and masters teams), (b) be able to read and respond to the Korean-language questionnaire, and (c) agree voluntarily to participate, with parental consent for athletes younger than 18 years. All questionnaire items were set as required in the online form, so every submitted questionnaire was complete, and no participant was excluded for missing data. Responses abandoned before submission are not recorded by the survey platform, and the number of athletes who received the invitation could not be determined because the link was distributed through coaches.

The sample comprised 50 high-school athletes (28.9%), 41 collegiate athletes (23.7%), and 82 athletes competing at the general level (47.4%). Participants were aged 15–63 years (M = 25.92, SD = 9.29); 20 participants were younger than 18 years. Playing experience averaged 6.77 years (SD = 6.38). The general level (*n* = 82) included adult club and masters players: 23 general-level participants had no National Sports Festival experience, 24 had less than 3 years of playing experience (one further response could not be classified), and the oldest participant (63 years) was an adult club player. Only 11 participants (6.4%) were female. Because the sex composition of the eligible population is unknown, we could not verify whether this proportion is representative, and the findings should not be generalized to female athletes. Detailed demographic characteristics are presented in Table 1.

### 2.2. Measures

All constructs were assessed with self-report items developed with reference to established instruments. Item wording was adapted for water polo athletes by the research team together with co-researchers familiar with competitive water polo. The items were written in Korean on the basis of the content of the original scales; they are therefore adapted rather than formally translated versions, and no back-translation, formal content-validity rating, or pilot test was conducted. Evidence of internal consistency and factorial structure was therefore obtained only in the present sample (Section 3.2 and Tables S2 and S3); this evidence describes how the adapted items functioned in this sample and does not demonstrate psychometric equivalence with the original instruments, as measurement invariance between the adapted and original versions was not tested. All items were rated on a 5-point agreement scale ranging from 1 (strongly disagree) to 5 (strongly agree), which differs from the original response formats (7-point scales for the BNSSS and SMS-II and a 5-point frequency scale for the ABQ). This single response format was adopted to maintain consistency across the online questionnaire, so that participants could respond to all items in the same format. We acknowledge, however, that this decision departs from the original instruments: reducing the number of response options for the BNSSS and SMS-II may affect score variability, and replacing the frequency format of the ABQ with an agreement format shifts the focus from how often burnout-related experiences occur to the extent to which athletes endorse them. Scores obtained in this study are therefore not directly comparable with those obtained with the original instruments or with published norms, and this is acknowledged as a limitation (Section 5). The full item wording is provided in Table S1.

#### 2.2.1. Basic Psychological Need Satisfaction

Basic psychological need satisfaction was measured with 20 items adapted from the Basic Needs Satisfaction in Sport Scale (BNSSS) [25], reflecting the three needs specified in Self-Determination Theory [10]: autonomy (6 items), competence (7 items), and relatedness (7 items) (for example, “I feel that my opinions are reflected to some extent in the training process” for autonomy). This three-subscale allocation differs from the original BNSSS, in which autonomy is represented by separate choice, volition, and internal perceived locus of causality subscales. Higher scores indicate greater satisfaction of the corresponding psychological need.

#### 2.2.2. Self-Determined Motivation

Self-determined motivation was assessed with 20 items developed with reference to the Sport Motivation Scale-II (SMS-II) [26] and tailored to the Korean water polo context. The items covered intrinsic motivation (3 items), identified regulation (3 items), integrated regulation (6 items), controlled regulation (4 items), and amotivation (4 items). The controlled-regulation subscale combined external and introjected content: three items referred to the expectations, disappointment, or recognition of other people, which corresponds to external regulation in the SMS-II, and one item referred to guilt or a sense of failure (introjected regulation). External regulation was therefore represented within this combined subscale rather than as a separate regulatory style. Item content was also not perfectly aligned with the assigned regulations: Q04 refers to interest in learning new skills, which is closer to intrinsic motivation, and Q07–Q08 refer to meaning and goals, which overlap with identified regulation (Table S1).

An adapted Relative Autonomy Index (RAI) was calculated to summarize the overall degree of self-determined motivation of the athletes. The weighting scheme followed the relative ordering of regulatory styles along the self-determination continuum and the quasi-simplex pattern described in prior motivation research [27,28,29,30,31,32,33]. Mean scores for each regulation were weighted as follows: intrinsic motivation (+3), integrated regulation (+2), identified regulation (+1), controlled regulation (−1), and amotivation (−2). The adapted RAI was calculated as 3(Intrinsic Motivation) + 2(Integrated Regulation) + 1(Identified Regulation) − 1(Controlled Regulation) − 2(Amotivation), with higher scores indicating a more self-determined motivational profile. In the present sample, RAI scores ranged from 1.50 to 27.00 (M = 18.44, SD = 5.56), and the reliability of the weighted composite, estimated from the subscale reliabilities and covariances, was 0.964. Because aggregation does not preserve the unique contribution of each regulation [33], alternative operationalizations of motivation were examined in sensitivity analyses (Section 3.6).

#### 2.2.3. Athlete Burnout

Athlete burnout was measured with 15 items adapted from the Athlete Burnout Questionnaire (ABQ) [14], assessing emotional/physical exhaustion, a reduced sense of accomplishment, and sport devaluation (5 items each). Higher scores indicate a greater degree of burnout.

#### 2.2.4. Dropout Intention

Dropout intention was assessed with 4 items based on those developed by Sarrazin et al. [34] and subsequently adapted by Quested et al. [18] to measure the intention of youth soccer players to discontinue participation in the following season. The items refer to having thought about quitting water polo in the near future, frequently considering whether to continue one’s water polo career, wishing to switch to another sport or activity, and a low likelihood of continuing water polo in the next season. They therefore concern discontinuing water polo participation rather than leaving a particular team, but they combine past thoughts, current uncertainty, a conditional preference, and a next-season expectation, so the scale is interpreted as a broad withdrawal-intention score rather than a specific next-season probability. Higher scores indicate a stronger intention to drop out. The composition, item allocation, and internal consistency reliability (Cronbach’s α) of all measurement instruments are summarized in Table 2.

### 2.3. Procedure

Following the Institutional Review Board’s approval, an official letter requesting cooperation was sent to the coaching staff of water polo teams nationwide, and the online questionnaire (Google Forms, Google LLC, Mountain View, CA, USA). was administered from 20 to 27 August 2026. The survey link, a QR code, or both were distributed through coaches, team officials, governing organizations, and athlete networks. On accessing the survey, participants viewed an information sheet describing the study purpose, procedures, expected duration, voluntary nature of participation, right to withdraw, and data-protection procedures. Participants aged 18 years or older provided online informed consent. For athletes under 18 years, a parent or legal guardian first provided consent, after which the athlete completed an age-appropriate assent form. Only participants with the required consent and assent could proceed to the questionnaire, which took approximately 7–10 min to complete.

No directly identifying information was included in the analytic dataset. Mobile phone numbers were collected solely in order to deliver the participation incentive and were not used in the statistical analyses, and the analytic dataset contained only general characteristics and responses to the study variables. Participants received a mobile gift voucher (5000 KRW) on completing the survey. The study was approved by the Institutional Review Board of Hanseo University (Approval No. HS26-0820-03, approved 20 August 2026).

### 2.4. Data Analysis

Descriptive statistics, Cronbach’s alpha, and zero-order correlations were computed with SPSS version 26.0(IBM Corp., Armonk, NY, USA). Convergent validity was evaluated with composite reliability (CR) and average variance extracted (AVE), and discriminant validity with the Fornell–Larcker criterion [35]. Maximum-likelihood (ML) estimation with the Wishart likelihood, as in AMOS, was used for the main analyses [36]. Confirmatory factor analysis (CFA) and structural equation modeling (SEM) were conducted in AMOS 23.0 (IBM Corp., Armonk, NY, USA), and all latent-variable models were also estimated in R 4.3.3 (R Foundation for Statistical Computing, Vienna, Austria) with lavaan 0.6-17 [37] and semTools 0.5-6 [38], with the original AMOS estimates retained as the primary results. The original AMOS output provides the principal structural path estimates and the reported total associations and their bootstrap intervals; the R bootstrap analysis provides the three specific indirect path products and their intervals. These results are distinguished by analysis source and are not treated as interchangeable. Because multivariate normality was not satisfied (Section 3.1), the main model was also estimated with robust ML (MLR) [39] and evaluated with the Bollen–Stine bootstrap [40]. MLR addresses non-normality under a continuous-indicator analysis; it is not a categorical-data estimator. Multivariate normality was assessed with Mardia’s coefficient, and multivariate outliers with Mahalanobis distance (*p* < 0.001).

#### 2.4.1. Measurement Specification

Item parcels were used for need satisfaction and burnout to obtain a parsimonious specification [41]. Before parceling, the unidimensionality of each subdimension was examined with single-factor CFAs. Within each subdimension, items were divided into two parcels, and parcel scores were calculated as item means. For all subdimensions except reduced accomplishment, odd- and even-numbered items formed the two parcels; for reduced accomplishment, the parcels were Q46 and Q48 versus Q47, Q49, and Q50 (allocation in Table S1). Need satisfaction and burnout were each specified as a first-order factor indicated by six parcels, and the residuals of the two parcels from the same subdimension were allowed to covary (six residual covariances). These residual covariances were added in the original analysis after inspection of modification indices. They represent subdimension-specific variance shared by parcels from the same subdimension. Because they were data-driven, the fit of the measurement model should not be regarded as an independent confirmation of its structure. Dropout intention was indicated by its four items, and self-determined motivation by the manifest RAI. An item-level model without parcels, with need satisfaction and burnout as second-order factors, was estimated as a sensitivity analysis.

#### 2.4.2. Discriminant Validity of Burnout and Dropout Intention

Because these constructs are conceptually close, their distinctiveness was evaluated with several criteria: the Fornell–Larcker criterion, the heterotrait–monotrait (HTMT) ratio [42], the 95% confidence interval of the latent correlation, and chi-square difference tests comparing the freely estimated correlation with models in which it was fixed at 1.0 and at 0.90 [43]. Competing measurement models (one factor; two factors with or without the within-subdimension residual covariances; four correlated factors) were compared, and the correlations of dropout intention with each burnout dimension were examined.

#### 2.4.3. Structural Model and Indirect Associations 

Model fit was evaluated with χ^2^, χ^2^/df, the Comparative Fit Index (CFI), the Tucker–Lewis Index (TLI), the Root Mean Square Error of Approximation (RMSEA), and the Standardized Root Mean Square Residual (SRMR); the Goodness-of-Fit Index (GFI) is reported for continuity. Indirect associations were tested with 5000 bootstrap resamples and 95% bias-corrected (BC) confidence intervals. The sequential association hypothesized in H6 (need satisfaction → self-determined motivation → burnout → dropout intention), and the association via burnout alone (need satisfaction → burnout → dropout intention) were examined as specific products of their constituent paths. The specified partial model also includes a motivation-only route (need satisfaction → self-determined motivation → dropout intention). All three specific indirect associations were therefore estimated separately so that their combined value could be compared with the total indirect association. These cross-sectional estimates describe statistical associations within the specified model, not temporal or causal mediation. The hypothesized model was compared with a more parsimonious model without the two direct paths to dropout intention and an alternative model in which dropout intention preceded burnout; such comparisons cannot establish causal ordering [44].

#### 2.4.4. Sensitivity Analyses and Sample Size

The robustness of the main findings was examined by (a) excluding sport devaluation from burnout; (b) specifying the three burnout dimensions as separate, correlated intervening variables; (c) replacing the RAI with a latent autonomous-motivation factor, with simultaneous latent autonomous, controlled, and amotivation factors, or with a unit-weighted motivation index; (d) estimating an item-level model; (e) adding age (or competitive division), sex, playing experience, national-team experience, and injury in the past year as covariates of motivation, burnout, and dropout intention (n = 171, because two playing-experience responses could not be converted); (f) excluding multivariate outliers and invariant responders; (g) restricting the general level to participants with National Sports Festival experience, or excluding general-level participants with less than 3 years of (or unclassifiable) playing experience, as proxies for excluding adult club and masters players; and (h) evaluating common method variance with Harman’s single-factor test and an unmeasured latent method construct (ULMC) [45]. No a priori sample-size calculation was performed. Statistical power was therefore evaluated post hoc with an RMSEA-based power analysis [46] and a Monte Carlo simulation (1000 replications of N = 173 generated from the estimated model) [47]. Statistical significance was set at *p* < 0.05.

## 3. Results

### 3.1. Preliminary Analyses

Before hypothesis testing, univariate normality was examined for the four composite study variables. Skewness values ranged from −0.76 to 0.41, and kurtosis values from −0.54 to 0.17, and item-level skewness (−1.98 to 0.90) and kurtosis (−0.91 to 5.24) were also within the conventional thresholds for ML estimation (|skewness| < 2, |kurtosis| < 7) [36]. Multivariate normality was not satisfied, however: Mardia’s multivariate kurtosis for the 17 SEM indicators was 442.26 (expected value 323.00; z = 30.86, *p* < 0.001). Eight multivariate outliers (Mahalanobis *p* < 0.001) and three participants who gave the same response to all 59 items were identified; they were retained in the main analyses and excluded in a sensitivity analysis. No item responses were missing. In Harman’s single-factor test, the first unrotated factor accounted for 36.2% of the item variance. Descriptive statistics and zero-order Pearson correlations among the four composite variables are presented in Table 3.

### 3.2. Measurement Model

A CFA of all 12 subscales (32 indicators: two parcels each for autonomy, competence, relatedness, integrated regulation, exhaustion, reduced accomplishment, and sport devaluation, and individual items for the remaining subscales; no residual covariances) showed broadly adequate but not uniformly strong fit: χ^2^ = 706.405, df = 398, *p* < 0.001, χ^2^/df = 1.775, CFI = 0.942, TLI = 0.928, RMSEA = 0.067, 90% CI [0.059, 0.075], SRMR = 0.052, although GFI (0.800) was low. An item-level 12-factor CFA (59 items) fitted less well (CFI = 0.838, TLI = 0.826, RMSEA = 0.076, 90% CI [0.072, 0.080], SRMR = 0.066), which suggests item-level misfit that parceling may conceal; an item-level three-factor CFA of need satisfaction also fitted only moderately (CFI = 0.898, TLI = 0.884, RMSEA = 0.097, SRMR = 0.065). All item loadings were significant (0.55–0.93), CR ranged from 0.835 to 0.935, and AVE from 0.482 to 0.794; the AVE for autonomy (0.482) was slightly below 0.50, although its CR exceeded 0.80 (Table S2). Single-factor CFAs provided mixed evidence: CFI and SRMR were generally favorable (CFI = 0.930–0.999, SRMR = 0.010–0.057), but several RMSEA values were elevated (Table S2). The three-item intrinsic and identified models were just-identified and could not provide a global-fit test of unidimensionality. These results offer limited support for parceling and do not establish item-level unidimensionality. Two features of the motivation measure should be noted (Table S3): intrinsic motivation and identified regulation were almost perfectly correlated (r = 0.98; HTMT = 0.99) and were therefore not empirically distinguishable in this sample, consistent with the intrinsic content of Q04, and controlled regulation was nearly unrelated to the autonomous regulations (r = 0.04–0.11) but positively related to amotivation (r = 0.44).

For SEM, basic psychological need satisfaction and athlete burnout were specified as latent factors indicated by six parcels each, with the six within-subdimension residual covariances described in Section 2.4; dropout intention was indicated by its four items, and self-determined motivation was the manifest RAI. The standardized residual correlations were 0.764 (competence), 0.731 (relatedness), 0.659 (exhaustion), 0.322 (reduced accomplishment), 0.540 (sport devaluation), and −0.029 (autonomy; the original AMOS covariance test gave *p* = 0.881). The standardized loadings, CR, and AVE are presented in Table 4.

Discriminant validity was evaluated with the Fornell–Larcker criterion, which was met for the comparisons involving basic psychological need satisfaction. The latent correlation between athlete burnout and dropout intention in the measurement model corresponding to the structural model was 0.852. It exceeded the square root of the AVE for both burnout (0.828) and dropout intention (0.807), so the criterion was not met for this pair.

Because of this overlap, the distinctiveness of burnout and dropout intention was examined further (Table 5 and Table S4). With the same within-subdimension residual covariances as in the structural model, the two-factor model fitted well (Model 1). Without them (Model 2), it fitted poorly, because subdimension-specific variance shared by parcels was left unmodeled; this poor fit therefore did not by itself indicate a failure to distinguish burnout from dropout intention. The one-factor model without residual covariances (Model 3) fitted worst, but that comparison combines differences in factor structure and residual specification. When the one-factor model included the same residual covariances as Model 1 (Model 3b), it still fitted clearly worse than the two-factor model, Δχ^2^(1) = 53.757, *p* < 0.001, which isolates the factor-structure difference.

In the focused two-factor model, the latent correlation between burnout and dropout intention was 0.851, 95% CI [0.792, 0.911]; the corresponding full measurement-model correlation was 0.852. Fixing it at 1.0 significantly worsened fit (a test equivalent to the Model 3b vs. Model 1 comparison above), but fixing it at 0.90 did not, Δχ^2^(1) = 2.957, *p* = 0.086. The HTMT ratio for burnout (15 items) and dropout intention was 0.836 (bootstrap 95% CI [0.749, 0.898]), below the conservative 0.85 threshold [42], although the upper limit exceeded it. Across burnout dimensions, the overlap with dropout intention was concentrated in sport devaluation: latent correlations were 0.670 [0.573, 0.767] for exhaustion, 0.778 [0.701, 0.856] for reduced accomplishment, and 0.836 [0.776, 0.896] for sport devaluation, with HTMT values of 0.688, 0.788, and 0.867, respectively. In an exploratory factor analysis of the sport-devaluation, amotivation, and dropout-intention items, the three item sets formed separate factors, and only one dropout item (Q59) cross-loaded on sport devaluation (0.33 vs. 0.37). Following Rönkkö and Cho [43], these results indicate that burnout and dropout intention were not statistically identical but strongly overlapping (a moderate discriminant-validity concern). Because the Fornell–Larcker criterion was not met, discriminant validity is not regarded as conclusively established, and the association is interpreted as a strong association between burnout-related experiences and dropout intention rather than as a relation between clearly distinct constructs.

### 3.3. Structural Model

The hypothesized structural model showed marginally acceptable fit: χ^2^ = 229.737, df = 108, *p* < 0.001, χ^2^/df = 2.127, CFI = 0.953, TLI = 0.941, RMSEA = 0.081, 90% CI [0.066, 0.095], SRMR = 0.049, and GFI = 0.866. The upper limit of the RMSEA confidence interval and the Bollen–Stine bootstrap test (*p* = 0.036) indicate some misfit. With robust ML estimation, fit indices were somewhat better (scaled χ^2^ = 171.275, CFI = 0.967, TLI = 0.958, RMSEA = 0.068, SRMR = 0.049), and all path estimates were unchanged. The structural estimates are presented in Table 6.

**Figure 2:**
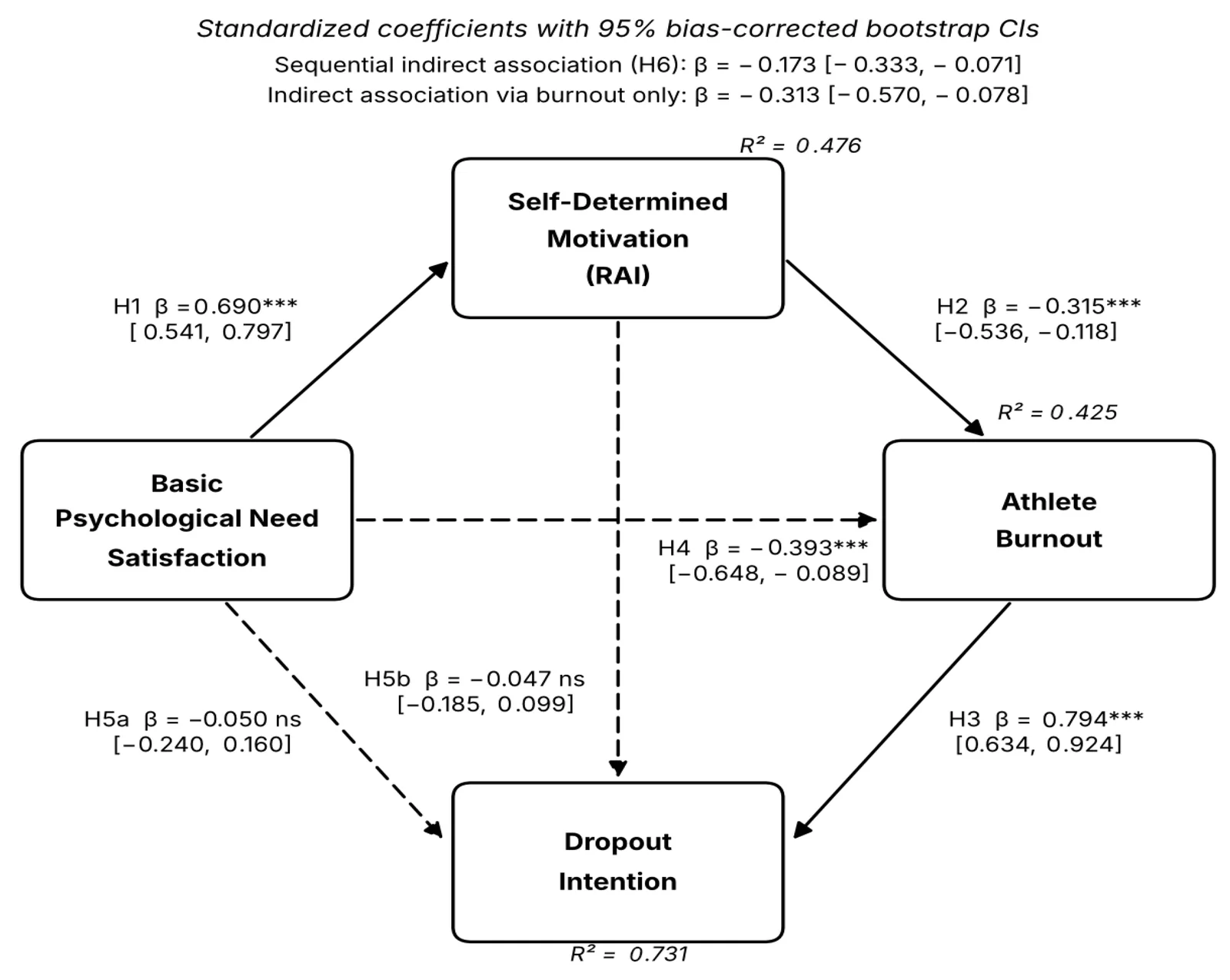
Standardized path coefficients for the tested structural model, with 95% bias-corrected bootstrap confidence intervals. Solid arrows denote the hypothesized sequential paths (H1–H3), which together constitute the sequential indirect association tested in H6, and dashed arrows denote the additional direct paths (H4–H5). The six within-subdimension residual covariances among parcels were included in estimation but are omitted from the figure. *** *p* < 0.001; ns = not significant.

H1–H4 were supported, whereas the two direct paths to dropout intention (H5a and H5b) were not significant, and athlete burnout was strongly associated with dropout intention (β = 0.794). The model accounted for 47.6% of the variance in self-determined motivation, 42.5% of that in athlete burnout, and 73.1% of that in dropout intention. Given the strong overlap between burnout and dropout intention (Section 3.2), the latter value partly reflects shared content between predictor and outcome and should not be read as evidence of high explanatory power. Figure 2 presents the standardized path coefficients.

### 3.4. Indirect Associations

Because the model contained more than one indirect route from need satisfaction to dropout intention, the sequential indirect association hypothesized in H6 (need satisfaction → self-determined motivation → burnout → dropout intention) and the association via burnout alone (need satisfaction → burnout → dropout intention) were estimated separately. Unstandardized estimates with 95% BC confidence intervals and standardized estimates are reported in Table 7.

The sequential indirect association specified in H6 was significant (B = −0.313, 95% BC CI [−0.667, −0.123], β = −0.173), as was the indirect association through burnout alone (B = −0.568, 95% BC CI [−1.026, −0.153], β = −0.313). The motivation-only indirect association was not significant (B = −0.058, 95% BC CI [−0.234, 0.141], β = −0.032). The total indirect association was B = −0.940 (95% BC CI [−1.355, −0.611], β = −0.517). The component estimates sum to approximately the total indirect estimate, allowing for rounding. The burnout-only route accounts for roughly 60% and the sequential route for roughly 33% of the unstandardized total indirect estimate; these descriptive proportions do not establish independent causal mechanisms. The indirect association from motivation to dropout intention through burnout was also significant (B = −0.041, 95% BC CI [−0.074, −0.015], β = −0.250). Thus, H6 was supported as a statistical association within the specified cross-sectional model, but the sequential route was not the only significant indirect route. Moreover, this support was conditional on the operationalization of motivation: H6 was supported with the adapted RAI and with a unit-weighted index, but not when a latent autonomous-motivation factor was used (Section 3.6).

### 3.5. Nested Model Comparison

To determine whether the two non-significant direct paths to dropout intention could be removed without a meaningful loss of fit, the hypothesized model (Model 1) was compared with a model without these direct paths (Model 2). An alternative model in which dropout intention preceded burnout (Model 3: need satisfaction → motivation → dropout intention → burnout, with the same number of parameters as Model 2) was also estimated. The fit indices are presented in Table 8.

Constraining the two direct paths to zero did not significantly worsen model fit, Δχ^2^(2) = 1.456, *p* = 0.483, and Model 2 returned slightly better CFI, TLI, RMSEA, AIC, and BIC values (Table 8). This indicates that the direct paths were not needed for model fit; it does not establish full mediation because the data are cross-sectional. The alternative ordering (Model 3) fitted somewhat worse than Model 2 (χ^2^ = 239.290 vs. 231.193 with equal df; AIC = 325.290 vs. 317.193). This comparison favours the hypothesized ordering only weakly: a model in which burnout and dropout intention merely covary has the same fit as Model 1, so the data cannot establish the direction of the burnout–dropout association.

### 3.6. Sensitivity Analyses

The burnout–dropout association remained strong across the sensitivity analyses summarized in Table 9 (selected full estimates in Table S5), whereas the sequential indirect association depended on the operationalization of motivation. The association between burnout and dropout intention remained strong when sport devaluation was excluded from burnout (β = 0.663, R^2^ = 0.640), in the item-level model, after adjustment for covariates (none of which was significantly associated with motivation, burnout, or dropout intention), after excluding outliers and invariant responders, when likely adult club and masters players were excluded (β = 0.801 and 0.800; sequential β = −0.186 and −0.223), and with robust estimation. The stability of the burnout–dropout coefficient in the item-level model should not, however, be taken as evidence of factorial validity: that model fitted poorly (CFI = 0.838, RMSEA = 0.088; Table 9), and stable structural coefficients do not resolve concerns about the measurement structure. When the three burnout dimensions were entered as separate correlated intervening variables, however, only sport devaluation retained a unique association with dropout intention (β = 0.570, 95% CI [0.342, 0.798]; reduced accomplishment β = 0.235, [−0.084, 0.554]; exhaustion β = −0.024, [−0.249, 0.201]); without sport devaluation, reduced accomplishment was strongly associated with dropout intention (β = 0.643, [0.374, 0.912]). The unconstrained ULMC model returned a negative method-factor variance (−0.006), indicating an inadmissible variance estimate. Its fit comparison and structural intervals are therefore not used as evidence against common method bias or as a robustness check. A constrained re-estimation with a non-negative method-factor variance placed the variance near its lower bound (approximately 0.0001) and yielded a similar burnout–dropout coefficient (β = 0.794). This boundary result does not rule out common method bias; conventional chi-square tests at a variance boundary are not straightforward to interpret.

The motivation results depended on the operationalization of self-determined motivation. With a unit-weighted index, the sequential indirect association remained significant (β = −0.183, 95% CI [−0.265, −0.102]). When the RAI was replaced by a latent autonomous-motivation factor, however, autonomous motivation was not associated with burnout, and the sequential indirect association was not significant (β = 0.018, 95% CI [−0.080, 0.117]). When autonomous, controlled, and amotivation factors were entered simultaneously, only amotivation was associated with burnout (β = 0.561, 95% CI [0.411, 0.710]; autonomous β = 0.095, *p* = 0.230; controlled β = −0.004, *p* = 0.949), and the sequential indirect association via amotivation and burnout was significant (β = −0.196, 95% CI [−0.284, −0.108]). Thus, the RAI–burnout association, and hence H6, was carried mainly by the amotivation component of the RAI (Table S6).

With respect to sample size, the RMSEA-based power of the test of close fit (H0: RMSEA = 0.05 vs. Ha: RMSEA = 0.08) was 0.93 for df = 108 and N = 173 [46]. In the Monte Carlo simulation, power was 0.91–1.00 for H1–H4 and 0.90 for the sequential indirect association, but only 0.10–0.11 for the small direct paths H5a and H5b (Table S7). These model-conditional, post hoc detection rates do not establish prospective sample-size adequacy or BC-bootstrap-test power. The non-significance of these direct paths should not be interpreted as evidence that they are zero.

## 4. Discussion

This study examined a theoretically specified pattern of associations among basic psychological need satisfaction, self-determined motivation, athlete burnout, and dropout intention in Korean water polo players. H1–H4 were supported, the direct paths specified in H5 were not significant, and the sequential indirect association proposed in H6 was significant. Need satisfaction was indirectly associated with dropout intention, most strongly via burnout alone. These findings are qualified by the strong overlap between burnout and dropout intention and by the dependence of H6 on the amotivation component of the RAI, as discussed below.

### 4.1. Basic Psychological Need Satisfaction and Self-Determined Motivation

Basic psychological need satisfaction showed a strong positive association with self-determined motivation (β = 0.690), which supports H1 and is consistent with the central proposition of self-determination theory (SDT) that satisfying autonomy, competence, and relatedness facilitates internalization and more autonomous forms of behavioural regulation [10,11]. The finding is consistent with prior sport research showing that athletes who experience greater psychological need satisfaction tend to report higher-quality, more self-determined motivation [12], and it extends that evidence to Korean water polo.

### 4.2. Psychological Need Satisfaction, Motivation, and Athlete Burnout

Both basic psychological need satisfaction and self-determined motivation were negatively associated with athlete burnout, which supports H2 and H4. These findings accord with previous evidence that psychological need satisfaction is negatively related to athlete burnout, both directly and in conjunction with the quality of athletes’ motivation [15]. The sensitivity analyses, however, qualify the support for H2. The association between the RAI and burnout was carried mainly by amotivation: when the motivational regulations were modeled separately, amotivation was strongly associated with burnout, whereas autonomous motivation showed no unique association once need satisfaction was taken into account. Moreover, intrinsic motivation and identified regulation could not be distinguished empirically, and controlled regulation was only weakly related to the other regulations. Accordingly, the support for H2 does not indicate a general protective role of autonomous motivation against burnout; rather, it highlights the specific importance of amotivation.

A direct association between need satisfaction and burnout persisted once self-determined motivation had been accounted for, which accords with the findings reported by De Francisco et al. [12]. One possible interpretation is that lower satisfaction of the needs for autonomy, competence, or relatedness is associated with burnout through experiences other than motivational internalization, such as perceptions of effectiveness, control, or support within the team. Because these experiences and need frustration were not measured, future research should examine whether team climate, perceived role security, or coach–athlete relational factors help to explain the residual association between need satisfaction and burnout.

It is also useful to consider how specific regulations might relate to the sequential pattern if analyzed separately. Theoretically, amotivation, which reflects a lack of perceived reasons for participating, is closest to the sport devaluation and withdrawal cognitions that characterize burnout and dropout intention, and in our data amotivation correlated 0.56–0.66 with the burnout dimensions and 0.64 with dropout intention. Intrinsic and other autonomous regulations would be expected to protect against exhaustion and reduced accomplishment mainly through enjoyment and perceived competence, which are closely tied to need satisfaction itself; this may explain why autonomous motivation showed no association with burnout beyond need satisfaction. Controlled regulation, which correlated positively with exhaustion (r = 0.31), may relate to burnout through pressure and obligation rather than through a lack of reasons. Part of the amotivation–burnout–dropout association may also reflect overlapping item content (e.g., questioning whether to continue). Future studies should therefore model the full set of regulations, including separate external and introjected regulation, with validated items.

### 4.3. Athlete Burnout and Dropout Intention

Athlete burnout showed a strong positive association with dropout intention, which supports H3. The finding is consistent with the sport commitment perspective, used here as a complementary interpretive lens, according to which withdrawal becomes more likely as the psychological and emotional costs of continued participation rise relative to its perceived benefits [13,16].

The magnitude of this association nonetheless requires careful interpretation. Burnout and dropout intention overlapped strongly (focused-model latent r = 0.851, 95% CI [0.792, 0.911]; full measurement-model r = 0.852). Although the alternative measurement specifications offer some evidence that the constructs are not identical, discriminant validity was not conclusively established (the Fornell–Larcker criterion was not met, and the upper limit of the HTMT confidence interval exceeded 0.85), particularly because sport devaluation was the only burnout dimension with a unique association with dropout intention. The burnout–dropout association remained substantial when sport devaluation was excluded (β = 0.663); thus, it cannot be attributed solely to that dimension, but the R^2^ of 0.731 should not be interpreted as evidence of clearly separable explanatory constructs. A possible late-stage withdrawal continuum and a shared negative evaluation of sport are competing interpretations that cannot be distinguished with these cross-sectional self-reports. The possibility that limited opportunities to change teams amplify this association in Korean water polo was not directly tested.

### 4.4. Direct Associations with Dropout Intention

Neither basic psychological need satisfaction nor self-determined motivation retained a significant direct association with dropout intention once athlete burnout had been included in the model, so H5 was not supported. This differs from previous evidence suggesting more direct protective associations between motivational variables and continued sport participation [17]. The Monte Carlo simulation showed that power to detect direct paths of the estimated size was low (0.10–0.11), so the absence of significant direct associations should not be interpreted as evidence that need satisfaction or motivation are unrelated to dropout intention independently of burnout.

One tentative explanation is that lower need satisfaction or motivation may not translate directly into withdrawal intention in a small and highly specialized sport, in which interpersonal ties, accumulated investment, and limited alternatives may sustain participation even when motivational quality is low; dropout intention may become more salient once such experiences are accompanied by burnout, particularly sport devaluation. Because these factors were not measured, this explanation remains speculative.

### 4.5. Sequential Indirect Associations

The specified sequential indirect association from basic psychological need satisfaction to dropout intention via self-determined motivation and burnout was significant (β = −0.173), supporting the statistical pattern hypothesized in H6. However, the association through burnout alone was larger; the result depended on how motivation was operationalized; and the RAI–burnout relation was attributable primarily to amotivation rather than to autonomous motivation considered separately (Section 4.2). In particular, when the RAI was replaced by a latent autonomous-motivation factor, the sequential indirect association was no longer significant, whereas the corresponding association via amotivation remained significant. H6 should therefore be regarded as supported only when motivation was operationalized as a composite index (the adapted RAI or a unit-weighted index), and not when autonomous motivation was modeled as a latent factor. These findings do not provide general support for a protective role of autonomous motivation; rather, their theoretical contribution lies in highlighting the specific importance of amotivation, that is, the absence of perceived reasons for participating, in relation to athlete burnout and, through burnout, to dropout intention. In this respect, the study extends previous water polo research [9] by considering need experiences, motivational quality, burnout, and dropout intention together, while the motivation-only route was not significant.

The sequential indirect association should not be read as evidence of a temporal or causal process: because all constructs were measured concurrently, the statistical ordering reflects the theoretically specified model rather than change demonstrated over time. Reverse or reciprocal associations are equally plausible. Athletes who already intend to leave water polo may evaluate their sport environment more negatively, report lower need satisfaction, endorse more amotivation, and report greater sport devaluation. Although a model in which dropout intention preceded burnout fitted slightly worse than the hypothesized model, an equivalent model in which burnout and dropout intention merely covaried fitted identically, so the fit of the hypothesized model does not establish its causal ordering [44].

### 4.6. Practical Implications

The strong association between burnout-related experiences and dropout intention suggests that attention to burnout, particularly sport devaluation and amotivation, may be relevant for coaches and practitioners in Korean water polo, where the competitive base is small [4,8]. Coaches shape many of the daily experiences through which athletes perceive autonomy, competence, and relatedness, and autonomy-supportive practices—providing meaningful rationales, acknowledging athletes’ perspectives, offering appropriate choice, and delivering competence-supportive feedback—are theoretically expected to support need satisfaction. Monitoring perceived training demands and recovery and discussing athletes’ reasons for participating may also be useful. These are theory-informed considerations derived from cross-sectional associations; whether such practices reduce burnout, amotivation, dropout intention, or actual dropout requires longitudinal and intervention research.

## 5. Limitations and Future Research

Several limitations concern measurement and construct distinctiveness. Burnout and dropout intention overlapped strongly (full measurement-model r = 0.852; focused-model r = 0.851, upper CI limit 0.911), particularly for sport devaluation, and their empirical distinctiveness remains uncertain despite sensitivity analyses. The adapted questionnaire items were not back-translated, formally evaluated for content validity, or pilot-tested. The use of a uniform 5-point agreement format, which differed from the original response formats of the BNSSS, SMS-II, and ABQ, further limits direct comparability with the original instruments and does not establish psychometric equivalence. The adapted RAI combined external and introjected regulation in a short controlled-regulation subscale, while its association with burnout was driven mainly by amotivation. Item parceling and data-driven within-subdimension residual covariances may also have obscured item-level misfit; the stability of structural coefficients across specifications does not resolve concerns about factorial validity or independently confirm the measurement structure. The study design and sample also limit interpretation. The cross-sectional, self-report design precludes conclusions about temporal or causal relationships, and reverse, or reciprocal associations remain plausible. Harman’s test cannot rule out common method bias, while the unmeasured latent method construct produced an inadmissible unconstrained solution and a boundary-constrained solution, limiting conclusions about shared-method variance. Recruitment through coaches and teams included only active athletes, potentially excluding those already disengaged or withdrawn. The predominantly male sample (93.6%) included athletes from different competitive levels, while club and masters membership was not directly recorded, limiting generalizability and subgroup comparisons. No a priori sample-size calculation was performed, and post hoc simulations cannot replace prospective power analysis, particularly for weak direct paths. Although sensitivity analyses supported the main associations, subgroup differences remain uncertain. Finally, dropout intention rather than actual withdrawal was measured. Future longitudinal, multi-source studies should assess actual retention, clarify temporal relationships, and validate the measurement structure in independent samples.

## 6. Conclusions

In this cross-sectional sample of 173 Korean water polo athletes, need satisfaction showed model-based indirect associations with dropout intention via burnout alone and sequentially via the adapted motivation index and burnout. Burnout-related experiences were strongly associated with dropout intention; however, given their substantial overlap—especially with sport devaluation—and the unmet Fornell–Larcker criterion, the discriminant validity of the two constructs was not conclusively established. The sequential association was not significant when autonomous motivation was modeled as a latent factor and appeared to reflect the specific role of amotivation rather than a general protective role of autonomous motivation. These results identify theoretically relevant, but not causal, associations that warrant replication with validated measures and longitudinal assessment of actual withdrawal.

## Figures and Tables

**Table 1:** Demographic characteristics of participants.

Variable	Category	n	%
Sex	Male	162	93.6
	Female	11	6.4
Age	19 and under	50	28.9
	20–23	42	24.3
	24–30	40	23.1
	31 and older	41	23.7
Division	High school	50	28.9
	Collegiate	41	23.7
	General	82	47.4
Position	Goalkeeper	19	11.0
	Center	33	19.1
	Driver	68	39.3
	Wing	18	10.4
	Utility	12	6.9
	Other	23	13.3
Playing experience	<3 years	62	36.3
	3 to <6 years	29	17.0
	≥6 years	80	46.8
National team experience	Yes	55	31.8
	No	118	68.2
Injury experience (past year)	Yes	69	39.9
	No	104	60.1
National games experience	Yes	129	74.6
	No	44	25.4
Total		173	100.0

Note. N = 173. Age: M = 25.92, SD = 9.29, range 15–63 years; age bands are shown for descriptive purposes. Division was recoded into three categories—high school, collegiate, and general (including adult club and masters players)—with the eight players who selected ‘Other’ (aged 20–48 years) combined with the general category because they were adult athletes outside high school and collegiate programmes. Position reflects the six response options in the questionnaire; ‘Other’ denotes athletes who did not select one of the five listed positions. Playing experience (total years as a competitive athlete; M = 6.77, SD = 6.38) was recoded with cut-points of <36, 36–71, and ≥72 months; two responses that could not be converted to months were excluded from that row (percentages based on n = 171).

**Table 2:** Composition and reliability of the measurement instruments.

Variable	Subdimension	Items	Source Scale	Cronbach’s α
Basic psychological need satisfaction	Autonomy	6 (Q21–Q26)	BNSSS [25]	0.850
	Competence	7 (Q27–Q33)	BNSSS [25]	0.934
	Relatedness	7 (Q34–Q40)	BNSSS [25]	0.931
Self-determined motivation	Intrinsic motivation	3 (Q01–Q03)	SMS-II [26]	0.918
	Integrated regulation	6 (Q07–Q12)	SMS-II [26]	0.932
	Identified regulation	3 (Q04–Q06)	SMS-II [26]	0.909
	Controlled regulation (external/introjected)	4 (Q13–Q16)	SMS-II [26]	0.865
	Amotivation	4 (Q17–Q20)	SMS-II [26]	0.920
Athlete burnout	Emotional/physical exhaustion	5 (Q41–Q45)	ABQ [14]	0.902
	Reduced sense of accomplishment	5 (Q46–Q50)	ABQ [14]	0.893
	Sport devaluation	5 (Q51–Q55)	ABQ [14]	0.923
Dropout intention	Single factor	4 (Q56–Q59)	Sarrazin et al. [34]; Quested et al. [18]	0.875

**Table 3:** Descriptive statistics and correlations among study variables.

Variable	M	SD	Skew	Kurt.	1	2	3	4
1. Basic psychological need satisfaction	3.90	0.62	0.02	−0.38	1			
2. Self-determined motivation (RAI)	18.44	5.56	−0.76	0.17	0.545 ***	1		
3. Athlete burnout	2.41	0.78	0.07	−0.40	−0.527 ***	−0.550 ***	1	
4. Dropout intention	2.23	0.90	0.41	−0.54	−0.461 ***	−0.528 ***	0.764 ***	1

Note. N = 173. RAI = Relative Autonomy Index (composite self-determination score). *** *p* < 0.001.

**Table 4:** Standardized loadings and reliability of the measurement model (structural-model constructs).

Construct	Indicator	Std. Loading	CR	AVE
Basic psychological need satisfaction	Autonomy parcel 1	0.81	0.885	0.567
	Autonomy parcel 2	0.92		
	Competence parcel 1	0.68		
	Competence parcel 2	0.69		
	Relatedness parcel 1	0.68		
	Relatedness parcel 2	0.71		
Athlete burnout	Exhaustion parcel 1	0.78	0.929	0.686
	Exhaustion parcel 2	0.79		
	Reduced-accomplishment parcel 1	0.87		
	Reduced-accomplishment parcel 2	0.82		
	Devaluation parcel 1	0.89		
	Devaluation parcel 2	0.81		
Dropout intention	Item 1 (Q56)	0.89	0.881	0.651
	Item 2 (Q57)	0.84		
	Item 3 (Q58)	0.74		
	Item 4 (Q59)	0.74		

Note. All loadings were significant at *p* < 0.001. CR = composite reliability; AVE = average variance extracted. Standardized loadings are reported directly from the AMOS standardized solution; CR and AVE were computed from these loadings following Fornell and Larcker [35]. The reported CR uses the conventional loading-based formula and does not incorporate the within-subdimension residual covariances; it should not be interpreted as a covariance-adjusted reliability estimate. The residuals of the two parcels within each subdimension were allowed to covary (Section 3.2). Covariance-adjusted CR estimates are separately reported in Supplementary Table S4 (need satisfaction 0.784; burnout 0.833).

**Table 5:** Comparison of Alternative Measurement Models for Athlete Burnout and Dropout Intention.

Model	χ^2^	df	χ^2^/df	CFI	TLI	RMSEA	SRMR
Model 1: Two factors, with within-subdimension residual covariances	52.264	31	1.69	0.986	0.980	0.063	0.037
Model 2: Two factors, without residual covariances	204.234	34	6.01	0.888	0.852	0.171	0.052
Model 3: One factor	283.184	35	8.09	0.837	0.790	0.203	0.067
Model 3b: One factor, with the same residual covariances as Model 1	106.021	32	3.31	0.951	0.931	0.116	0.051
Model 4: Four correlated factors	35.777	29	1.23	0.995	0.993	0.037	0.023

Note. Models 1 and 2 specify athlete burnout and dropout intention as separate correlated factors, with (Model 1, as in the structural model; which can be represented by a second-order specification only under matching constraints and an admissible solution) or without (Model 2) the three within-subdimension residual covariances among burnout parcels. Model 3 combines all indicators under one factor without the residual covariances included in Model 1; because it differs from Model 1 in both factor structure and residual specification, no chi-square difference test between these two models is interpreted. Model 3b is a one-factor model with the same three residual covariances as Model 1 and is nested within it (Model 3b vs. Model 1: Δχ^2^(1) = 53.757, *p* < 0.001). Model 4 specifies exhaustion, reduced accomplishment, sport devaluation, and dropout intention as four correlated first-order factors. SRMR = standardized root mean square residual.

**Table 6:** Structural path estimates.

Path	B	SE	C.R.	*p*	β [95% BC CI]	Result
H1. BPNS → Self-determined motivation	7.622	0.891	8.557	<0.001	0.690 [0.541, 0.797]	Supported
H2. Self-determined motivation → Burnout	−0.040	0.012	−3.333	<0.001	−0.315 [−0.536, −0.118]	Supported
H3. Burnout → Dropout intention	1.022	0.115	8.853	<0.001	0.794 [0.634, 0.924]	Supported
H4. BPNS → Burnout	−0.556	0.146	−3.815	<0.001	−0.393 [−0.648, −0.089]	Supported
H5a. BPNS → Dropout (direct)	−0.091	0.152	−0.600	0.549	−0.050 [−0.240, 0.160]	Not supported
H5b. SDM → Dropout (direct)	−0.008	0.013	−0.611	0.541	−0.047 [−0.185, 0.099]	Not supported

Note. N = 173. B = unstandardized regression coefficient; SE = standard error; C.R. = critical ratio; β = standardized regression coefficient with 95% bias-corrected bootstrap confidence interval (5000 resamples, lavaan). B, SE, C.R., and *p* are the original AMOS maximum-likelihood estimates. BPNS = basic psychological need satisfaction; SDM = self-determined motivation. Squared multiple correlations (R^2^): self-determined motivation = 0.476; athlete burnout = 0.425; dropout intention = 0.731. H2 and the sequential association tested in H6 depended on the operationalization of motivation (Section 3.6), and H3 should be interpreted in light of the strong overlap between burnout and dropout intention (Section 3.2).

**Table 7:** Bootstrapped direct, indirect, and total associations with dropout intention.

Path	Component	B	95% BC CI	β	*p*
BPNS → SDM → Burnout → Dropout	Specific sequential indirect (H6)	−0.313	[−0.667, −0.123]	−0.173	0.027
BPNS → Burnout → Dropout	Specific indirect via burnout	−0.568	[−1.026, −0.153]	−0.313	0.009
BPNS → Dropout	Direct	−0.091	[−0.504, 0.277]	−0.050	0.645
BPNS → Dropout	Total indirect	−0.940	[−1.355, −0.611]	−0.517	<0.001
BPNS → Dropout	Total	−1.031	[−1.464, −0.693]	−0.567	<0.001
SDM → Dropout	Direct	−0.008	[−0.031, 0.017]	−0.047	0.528
SDM → Dropout	Indirect (via Burnout)	−0.041	[−0.074, −0.015]	−0.250	0.008
SDM → Dropout	Total	−0.049	[−0.082, −0.017]	−0.297	0.002
BPNS → SDM → Dropout	Specific indirect via motivation	−0.058	[−0.234, 0.141]	−0.032	0.533

Note. N = 173; 5000 bootstrap resamples. B = unstandardized estimate; β = standardized estimate; BC CI = bias-corrected 95% confidence interval for B; BPNS = basic psychological need satisfaction; SDM = self-determined motivation (adapted RAI). Total associations and their BC CIs are from AMOS; specific indirect associations and their BC CIs are from R (lavaan). Reported *p*-values for the R estimates use bootstrap-standard-error normal approximations; indirect associations are interpreted primarily from their BC CIs.

**Table 8:** Comparison of the hypothesized model with a model without direct paths and an alternative ordering.

Model	χ^2^	df	CFI	TLI	RMSEA	AIC	BIC
Model 1: Hypothesized model (direct paths H5a, H5b estimated)	229.737	108	0.953	0.941	0.081	319.737	461.635
Model 2: Without direct paths (H5a, H5b fixed to 0)	231.193	110	0.954	0.943	0.080	317.193	452.784
Model 3: Alternative ordering (dropout intention → burnout)	239.290	110	0.951	0.939	0.083	325.290	460.882
Difference (Model 2 − Model 1)	Δχ^2^ = 1.456	Δdf = 2					

Note. N = 173. Model 1 is the structural model reported in Table 6. AIC and BIC are computed as χ^2^ + 2q and χ^2^ + q·ln(N), where q is the number of free parameters (as in AMOS).

**Table 9:** Summary of sensitivity analyses (standardized estimates).

Model	Burnout → DI	H6 β	95% CI	R^2^ (DI)	CFI	RMSEA	SRMR
Main model (ML)	0.794	−0.173	[−0.276, −0.069]	0.731	0.953	0.081	0.049
Robust ML (MLR)	0.794	−0.173	[−0.301, −0.044]	0.731	0.967	0.068	0.049
Burnout without sport devaluation	0.663	−0.135	[−0.226, −0.043]	0.640	0.957	0.082	0.046
Item-level model (no parcels)	0.806	−0.174	[−0.271, −0.077]	0.738	0.838	0.088	0.072
Covariates: age, sex, experience, national team, injury (n = 171)	0.771	−0.154	[−0.257, −0.050]	0.738	0.933	0.076	0.055
Covariates: division instead of age (n = 171)	0.778	−0.146	[−0.252, −0.040]	0.734	0.929	0.076	0.055
Excluding outliers and invariant responders (n = 162)	0.855	−0.161	[−0.252, −0.070]	0.785	0.956	0.081	0.051
Unit-weighted motivation index	0.777	−0.183	[−0.265, −0.102]	0.735	0.951	0.083	0.050
Latent autonomous motivation (AM)	0.815	0.018	[−0.080, 0.117]	0.727	0.951	0.079	0.049
AM, controlled, and amotivation factors (via amotivation)	0.715	−0.196	[−0.284, −0.108]	0.744	0.923	0.079	0.066
General level restricted to National Sports Festival participants (n = 150)	0.801	−0.186	[−0.301, −0.071]	0.739	0.955	0.081	0.048
Excluding general-level players with <3 years’ experience (n = 148)	0.800	−0.223	[−0.350, −0.096]	0.736	0.942	0.090	0.056

Note. Burnout → DI = standardized path from burnout to dropout intention; H6 β = standardized sequential indirect association (H6 or its analogue) with 95% CI (delta method); fit indices are for each model (robust indices for MLR). The last two models exclude likely adult club and masters players from the general level (proxies: no National Sports Festival experience; less than 3 years of playing experience). In the covariate models, self-determined motivation, burnout, and dropout intention were regressed on the covariates. The unconstrained ULMC row was removed because its method-factor variance was negative. DI = dropout intention; AM = autonomous motivation; ULMC = unmeasured latent method construct; MLR = robust maximum likelihood.

## Data Availability

The data used and analyzed during the current study are available from the corresponding author upon reasonable request. The data are not publicly available due to privacy and ethical considerations, as the study included minor participants and involved a relatively small and specific population of water polo players, which may increase the risk of indirect identification.

## Supplementary Materials

The following supporting information can be downloaded at: https://www.mdpi.com/article/10.3390/healthcare14213748/s1. Table S1: Item wording (Korean original and English translation), parcel allocation, and within-subdimension residual covariances. Table S2: Item-level 12-factor CFA: standardized loadings, reliability, and single-factor CFAs. Table S3: Latent correlations and HTMT ratios among the 12 subscales. Table S4: Discriminant validity of athlete burnout and dropout intention. Table S5: Standardized structural estimates in selected sensitivity models. Table S6: Motivation decomposition: autonomous, controlled, and amotivation factors. Table S7: Statistical power.

## Abbreviations

The following abbreviations are used in this manuscript:

SDT	Self-Determination Theory
BPNS	Basic psychological need satisfaction
SDM	Self-determined motivation
RAI	Relative Autonomy Index
BNSSS	Basic Needs Satisfaction in Sport Scale
SMS-II	Sport Motivation Scale-II
ABQ	Athlete Burnout Questionnaire
SEM	Structural equation modeling
CFA	Confirmatory factor analysis
CFI	Comparative Fit Index
TLI	Tucker–Lewis Index
RMSEA	Root Mean Square Error of Approximation
GFI	Goodness-of-Fit Index
AVE	Average variance extracted
CR	Composite reliability
BC CI	Bias-corrected confidence interval
HTMT	Heterotrait–monotrait ratio
SRMR	Standardized root mean square residual
MLR	Robust maximum likelihood
ULMC	Unmeasured latent method construct
