# Supplementary material for: Psychological Need Satisfaction, Self-Determined Motivation, Burnout, and Dropout Intention in Korean Water Polo Athletes: A Cross-Sectional Structural Equation Model
Source: Healthcare (Basel). 2026 Nov 3;14(21):3748. doi: 10.3390/healthcare14213748 (PMC13762539; doi:10.3390/healthcare14213748)
Supplement: Supplementary file 1 [file healthcare-14-03748-s001.zip › healthcare-4597455-supplementary.pdf]

Supplementary Materials

Psychological Need Satisfaction, Self-Determined Motivation, Burnout, and Dropout Intention in Korean Water Polo Athletes: A Cross-Sectional Structural Equation Model

All latent-variable analyses were conducted in R 4.3.3 with lavaan 0.6-17 and semTools (ML with Wishart likelihood unless otherwise noted); N = 173 unless otherwise noted.

Table S1. Item wording (Korean original and English translation).

Item	Construct / subscale	Korean (administered)	English translation
Q01	Self-determined motivation / Intrinsic motivation	나는 수구를 하는 것이 즐겁기 때문에 참여한다.	I participate in water polo because it is enjoyable.
Q02	Self-determined motivation / Intrinsic motivation	나는 수구 기술을 배우고 익히는 과정이 재미있기 때문에 참여한다.	I participate because the process of learning and mastering water polo skills is fun.
Q03	Self-determined motivation / Intrinsic motivation	나는 수구 경기나 훈련에 몰입할 때 만족감을 느끼기 때문에 참여한다.	I participate because I feel satisfaction when I am absorbed in water polo games or training.
Q04	Self-determined motivation / Identified regulation	나는 수구를 통해 새로운 기술이나 전략을 배우는 것이 흥미롭다.	I find it interesting to learn new skills or strategies through water polo.
Q05	Self-determined motivation / Identified regulation	나는 수구가 나의 성장에 도움이 된다고 생각하기 때문에 참여한다.	I participate because I think water polo helps my personal growth.
Q06	Self-determined motivation / Identified regulation	나는 수구를 통해 체력, 기술, 정신력을 발전시킬 수 있다고 생각한다.	I think I can develop my fitness, skills, and mental strength through water polo.
Q07	Self-determined motivation / Integrated regulation	나는 수구 선수로 활동하는 것이 나에게 의미 있는 일이라고 생각한다.	I think that being a water polo athlete is meaningful to me.
Q08	Self-determined motivation / Integrated regulation	나는 수구가 나의 목표를 이루는 데 도움이 된다고 생각한다.	I think water polo helps me achieve my goals.
Q09	Self-determined motivation / Integrated regulation	수구는 나의 삶에서 중요한 부분이라고 생각한다.	I think water polo is an important part of my life.
Q10	Self-determined motivation / Integrated regulation	수구 선수로 활동하는 것은 내가 추구하는 가치와 잘 맞는다.	Being a water polo athlete fits well with the values I pursue.
Q11	Self-determined motivation / Integrated regulation	수구는 내가 어떤 사람인지를 표현하는 활동 중 하나라고 생각한다.	Water polo is one of the activities that expresses who I am.
Q12	Self-determined motivation / Integrated regulation	수구를 계속하는 것은 내가 선택한 삶의 방식과 관련이 있다.	Continuing water polo is related to the way of life I have chosen.
Q13	Self-determined motivation / Controlled regulation (external/introjected)	나는 주변 사람들의 기대 때문에 수구를 계속해야 한다고 느낀다.	I feel that I must continue water polo because of the expectations of people around me.

Q14	Self-determined motivation / Controlled regulation (external/introjected)	나는 수구를 그만두면 주변 사람들이 실망할 것 같아서 계속한다.	I continue water polo because the people around me would be disappointed if I quit.
Q15	Self-determined motivation / Controlled regulation (external/introjected)	나는 인정받거나 좋은 평가를 받기 위해 수구에 참여한다.	I participate in water polo to be recognized or to receive good evaluations.
Q16	Self-determined motivation / Controlled regulation (external/introjected)	나는 수구를 그만두면 죄책감이나 실패감을 느낄 것 같아서 계속한다.	I continue water polo because I would feel guilty or like a failure if I quit.
Q17	Self-determined motivation / Amotivation	나는 요즘 내가 왜 수구를 계속하고 있는지 잘 모르겠다.	These days, I am not sure why I keep playing water polo.
Q18	Self-determined motivation / Amotivation	나는 수구를 계속해야 하는 이유가 예전보다 분명하지 않다.	The reasons why I should continue water polo are less clear than before.
Q19	Self-determined motivation / Amotivation	나는 수구를 통해 무엇을 얻고 있는지 잘 모르겠다.	I am not sure what I am gaining from water polo.
Q20	Self-determined motivation / Amotivation	나는 앞으로도 수구를 계속해야 할지 의문이 든다.	I wonder whether I should continue water polo in the future.
Q21	Basic psychological need satisfaction / Autonomy	나는 수구 훈련에 참여할 때 내가 스스로 선택하고 있다는 느낌을 받는다.	When I take part in water polo training, I feel that I am making my own choices.
Q22	Basic psychological need satisfaction / Autonomy	나는 수구를 억지로 하는 것이 아니라 내가 원해서 하고 있다고 느낀다.	I feel that I play water polo because I want to, not because I am forced to.
Q23	Basic psychological need satisfaction / Autonomy	나는 훈련 과정에서 내 의견이 어느 정도 반영된다고 느낀다.	I feel that my opinions are reflected to some extent in the training process.
Q24	Basic psychological need satisfaction / Autonomy	나는 수구를 계속하는 이유가 나 자신에게 있다고 느낀다.	I feel that the reasons for continuing water polo lie within myself.
Q25	Basic psychological need satisfaction / Autonomy	나는 수구 훈련과 경기에서 스스로 결정할 수 있는 부분이 있다고 느낀다.	I feel that there are aspects of water polo training and games that I can decide for myself.
Q26	Basic psychological need satisfaction / Autonomy	나는 수구 선수로 생활하는 것이 내 가치와 잘 맞는다고 느낀다.	I feel that living as a water polo athlete fits well with my values.
Q27	Basic psychological need satisfaction / Competence	나는 수구 기술을 점점 더 잘 수행하고 있다고 느낀다.	I feel that I am performing water polo skills better and better.
Q28	Basic psychological need satisfaction / Competence	나는 훈련과 경기에서 요구되는 역할을 잘 해낼 수 있다고 느낀다.	I feel that I can successfully fulfil the roles required in training and games.
Q29	Basic psychological need satisfaction / Competence	나는 수구 선수로서 필요한 능력을 갖추고 있다고 느낀다.	I feel that I have the abilities needed as a water polo athlete.
Q30	Basic psychological need satisfaction / Competence	나는 어려운 훈련이나 경기 상황에서도 잘 대응할 수 있다고 느낀다.	I feel that I can cope well even in difficult training or game situations.

Q31	Basic psychological need satisfaction / Competence	나는 수구에서 실력이 향상되고 있다고 느낀다.	I feel that my water polo skills are improving.
Q32	Basic psychological need satisfaction / Competence	나는 팀에 경기력 측면에서 도움이 되는 선수라고 느낀다.	I feel that I am a player who contributes to my team's performance.
Q33	Basic psychological need satisfaction / Competence	나는 수구 경기에서 중요한 상황을 해결할 수 있다는 자신감이 있다.	I am confident that I can resolve serious situations in water polo games.
Q34	Basic psychological need satisfaction / Relatedness	나는 팀 동료들과 가깝게 연결되어 있다고 느낀다.	I feel closely connected with my teammates.
Q35	Basic psychological need satisfaction / Relatedness	나는 우리 팀 안에서 소속감을 느낀다.	I feel a sense of belonging in my team.
Q36	Basic psychological need satisfaction / Relatedness	나는 팀 동료들과 서로 신뢰하고 있다고 느낀다.	I feel that my teammates and I trust each other.
Q37	Basic psychological need satisfaction / Relatedness	나는 지도자와 팀 동료들로부터 존중받고 있다고 느낀다.	I feel respected by my coaches and teammates.
Q38	Basic psychological need satisfaction / Relatedness	나는 팀 안에서 나의 존재가 중요하다고 느낀다.	I feel that my presence matters within the team.
Q39	Basic psychological need satisfaction / Relatedness	나는 수구를 통해 의미 있는 인간관계를 형성하고 있다고 느낀다.	I feel that I am forming meaningful relationships through water polo.
Q40	Basic psychological need satisfaction / Relatedness	나는 팀 안에서 정서적으로 지지받고 있다고 느낀다.	I feel emotionally supported within the team.
Q41	Athlete burnout / Emotional/physical exhaustion	나는 수구 훈련과 경기 때문에 몸과 마음이 지쳐 있다고 느낀다.	I feel physically and mentally exhausted because of water polo training and games.
Q42	Athlete burnout / Emotional/physical exhaustion	나는 수구를 계속하기에는 에너지가 부족하다고 느낄 때가 많다.	I often feel that I lack the energy to continue water polo.
Q43	Athlete burnout / Emotional/physical exhaustion	나는 훈련이나 경기를 생각하면 피로감이 먼저 느껴진다.	When I think about training or games, I feel fatigued first.
Q44	Athlete burnout / Emotional/physical exhaustion	나는 수구로 인해 정서적으로 소진되어 있다고 느낀다.	I feel emotionally drained because of water polo.
Q45	Athlete burnout / Emotional/physical exhaustion	나는 훈련 후 회복이 충분히 되지 않는다고 느낀다.	I feel that I do not recover sufficiently after training.
Q46	Athlete burnout / Reduced sense of accomplishment	나는 예전만큼 수구에서 성취감을 느끼지 못한다.	I do not feel as much accomplishment in water polo as I used to.
Q47	Athlete burnout / Reduced sense of accomplishment	나는 수구 선수로서 충분히 발전하고 있는지 의문이 든다.	I doubt whether I am developing sufficiently as a water polo athlete.

Q48	Athlete burnout / Reduced sense of accomplishment	나는 훈련을 해도 기대만큼 실력이 좋아지지 않는다고 느낀다.	I feel that my skills do not improve as much as expected even when I train.
Q49	Athlete burnout / Reduced sense of accomplishment	나는 수구에서 내가 잘하고 있다는 느낌이 줄어들었다.	My feeling that I am doing well in water polo has decreased.
Q50	Athlete burnout / Reduced sense of accomplishment	나는 수구 선수로서의 목표를 이루기 어렵다고 느낀다.	I feel that it is difficult to achieve my goals as a water polo athlete.
Q51	Athlete burnout / Sport devaluation	나는 수구가 예전만큼 중요하게 느껴지지 않는다.	Water polo does not feel as important as it used to.
Q52	Athlete burnout / Sport devaluation	나는 수구에 대한 흥미가 줄어들었다고 느낀다.	I feel that my interest in water polo has decreased.
Q53	Athlete burnout / Sport devaluation	나는 수구 선수 생활의 의미를 자주 의심하게 된다.	I often doubt the meaning of my career as a water polo athlete.
Q54	Athlete burnout / Sport devaluation	나는 수구를 위해 많은 노력을 하는 것이 가치 있는 일인지 고민한다.	I wonder whether putting a lot of effort into water polo is worthwhile.
Q55	Athlete burnout / Sport devaluation	나는 수구에 대한 애정이나 열정이 예전보다 낮아졌다고 느낀다.	I feel that my affection or passion for water polo is lower than before.
Q56	Dropout intention / Dropout intention	나는 가까운 시일 내에 수구를 그만둘 생각을 한 적이 있다.	I have thought about quitting water polo in the near future.
Q57	Dropout intention / Dropout intention	나는 앞으로 수구 선수 생활을 계속할지 자주 고민한다.	I often consider whether to continue my career as a water polo athlete.
Q58	Dropout intention / Dropout intention	기회가 된다면 수구가 아닌 다른 종목이나 활동으로 전환하고 싶다.	If I had the chance, I would like to switch to a sport or activity other than water polo.
Q59	Dropout intention / Dropout intention	나는 다음 시즌에도 수구를 계속할 가능성이 낮다고 생각한다.	I think it is unlikely that I will continue water polo next season.

Note. All items were rated on a 5-point scale (1 = strongly disagree, 5 = strongly agree). Items were adapted by the research team with reference to the SMS-II, BNSSS, ABQ, and the dropout-intention items of Sarrazin et al. and Quested et al.; English translations are provided for the reader and were not administered. Q13–Q15 reflect external regulation and Q16 introjected regulation.

Table S1 (continued). Parcel allocation and within-subdimension residual covariances in the structural model.

**Subdimension**	**Parcel 1**	**Parcel 2**	**Residual correlation (standardized)**
Autonomy	Q21, Q23, Q25	Q22, Q24, Q26	−.029 (p = .881; AMOS)
Competence	Q27, Q29, Q31, Q33	Q28, Q30, Q32	.764 (p < .001)
Relatedness	Q34, Q36, Q38, Q40	Q35, Q37, Q39	.731 (p < .001)
Integrated regulation (12-factor CFA only)	Q07, Q09, Q11	Q08, Q10, Q12	—
Emotional/physical exhaustion	Q41, Q43, Q45	Q42, Q44	.659 (p < .001)

Reduced sense of accomplishment	Q46, Q48	Q47, Q49, Q50	.322 (p < .001)
Sport devaluation	Q51, Q53, Q55	Q52, Q54	.540 (p < .001)

Note. Parcel scores are item means. The six residual covariances were added in the original analysis after inspection of modification indices; a second-order representation may capture similar subdimension-specific covariance only under matching constraints and an admissible solution; unconditional statistical equivalence is not assumed. The reduced-accomplishment allocation departs from the odd/even pattern used for the other subdimensions.

Table S2. Item-level 12-factor CFA: standardized loadings, reliability, and single-factor CFAs.

Subscale	Items	Standardized loadings (12-factor CFA)	α	CR	AVE	Single-factor CFA
Intrinsic motivation	Q01–Q03	Q01 .83, Q02 .93, Q03 .92	.918	.922	.794	3 items (just-identified)
Identified regulation	Q04–Q06	Q04 .92, Q05 .85, Q06 .88	.909	.917	.776	3 items (just-identified)
Integrated regulation	Q07–Q12	Q07 .89, Q08 .85, Q09 .88, Q10 .84, Q11 .77, Q12 .78	.932	.931	.698	$\chi^2(9)$ = 19.09, CFI = .987, RMSEA = .081, SRMR = .021
Controlled regulation (external/introjected)	Q13–Q16	Q13 .77, Q14 .91, Q15 .65, Q16 .81	.865	.872	.630	$\chi^2(2)$ = 9.33, CFI = .979, RMSEA = .146, SRMR = .028
Amotivation	Q17–Q20	Q17 .85, Q18 .87, Q19 .89, Q20 .85	.920	.924	.747	$\chi^2(2)$ = 2.58, CFI = .999, RMSEA = .041, SRMR = .010
Autonomy	Q21–Q26	Q21 .55, Q22 .69, Q23 .60, Q24 .81, Q25 .73, Q26 .85	.850	.835	.482	$\chi^2(9)$ = 39.61, CFI = .930, RMSEA = .141, SRMR = .057
Competence	Q27–Q33	Q27 .76, Q28 .87, Q29 .84, Q30 .81, Q31 .79, Q32 .82, Q33 .85	.934	.935	.673	$\chi^2(14)$ = 55.78, CFI = .955, RMSEA = .132, SRMR = .040
Relatedness	Q34–Q40	Q34 .83, Q35 .75, Q36 .85, Q37 .81, Q38 .78, Q39 .82, Q40 .88	.931	.934	.666	$\chi^2(14)$ = 39.98, CFI = .971, RMSEA = .104, SRMR = .029
Emotional/physical exhaustion	Q41–Q45	Q41 .70, Q42 .84, Q43 .87, Q44 .90, Q45 .74	.902	.904	.653	$\chi^2(5)$ = 13.31, CFI = .985, RMSEA = .098, SRMR = .024
Reduced sense of accomplishment	Q46–Q50	Q46 .76, Q47 .85, Q48 .87, Q49 .80, Q50 .71	.893	.909	.640	$\chi^2(5)$ = 22.54, CFI = .966, RMSEA = .143, SRMR = .035
Sport devaluation	Q51–Q55	Q51 .81, Q52 .89, Q53 .90, Q54 .77, Q55 .85	.923	.928	.711	$\chi^2(5)$ = 8.39, CFI = .995, RMSEA = .063, SRMR = .017
Dropout intention	Q56–Q59	Q56 .89, Q57 .83, Q58 .75, Q59 .74	.875	.889	.654	$\chi^2(2)$ = 8.78, CFI = .982, RMSEA = .140, SRMR = .025

Note. 12-factor item-level CFA: $\chi^2(1586)$ = 3144.139, CFI = .838, TLI = .826, RMSEA = .076, 90% CI [.072, .080], SRMR = .066. The parcel-level 12-factor CFA used in the main text: $\chi^2(398)$ = 706.405, CFI = .942, TLI = .928, RMSEA = .067, SRMR = .052. Item-level three-factor CFA of need satisfaction: $\chi^2(167)$ = 437.897, CFI = .898, TLI = .884, RMSEA = .097, SRMR = .065. All loadings p < .001.

Table S3. Latent correlations (below diagonal) and HTMT ratios (above diagonal) among the 12 subscales.

	IM	IDEN	INTEG	CTRL	AMOT	AUT	COM	REL	EE	RA	DEV	DI

IM	1	.99	.86	.07	.36	.63	.46	.40	.24	.35	.31	.35
IDEN	.98	1	.89	.09	.36	.61	.36	.39	.27	.34	.32	.36
INTEG	.87	.88	1	.11	.34	.66	.48	.47	.35	.40	.34	.34
CTRL	.04	.04	.11	1	.42	.10	.17	.07	.33	.12	.22	.26
AMOT	−.35	−.36	−.34	.44	1	.47	.26	.32	.61	.63	.71	.71
AUT	.61	.58	.66	−.06	−.52	1	.73	.73	.53	.51	.54	.58
COM	.45	.35	.47	.15	−.25	.75	1	.66	.32	.50	.35	.38
REL	.41	.38	.46	.05	−.32	.74	.67	1	.40	.48	.53	.43
EE	−.23	−.26	−.34	.35	.60	−.53	−.33	−.42	1	.85	.76	.69
RA	−.35	−.35	−.40	.17	.60	−.49	−.49	−.46	.83	1	.84	.79
DEV	−.31	−.33	−.35	.26	.71	−.56	−.35	−.52	.77	.79	1	.87
DI	−.33	−.34	−.33	.26	.70	−.58	−.35	−.42	.68	.73	.84	1

Note. IM = intrinsic motivation; IDEN = identified regulation; INTEG = integrated regulation; CTRL = controlled regulation (external/introjected); AMOT = amotivation; AUT = autonomy; COM = competence; REL = relatedness; EE = emotional/physical exhaustion; RA = reduced sense of accomplishment; DEV = sport devaluation; DI = dropout intention. Square roots of AVE: IM .891, IDEN .881, INTEG .835, INTRO .794, AMOT .864, AUT .694, COM .820, REL .816, EE .808, RA .800, DEV .843, DI .809.

Table S4. Discriminant validity of athlete burnout and dropout intention.

Analysis	Result
Model 1: two factors, with within-subdimension residual covariances (as in the structural model)	$\chi^2(31) = 52.264$, CFI = .986, TLI = .980, RMSEA = .063 [.031, .092], SRMR = .037
Model 2: two factors, without residual covariances	$\chi^2(34) = 204.234$, CFI = .888, TLI = .852, RMSEA = .171 [.148, .194], SRMR = .052
Model 3: one factor	$\chi^2(35) = 283.184$, CFI = .837, TLI = .790, RMSEA = .203 [.181, .225], SRMR = .067
Model 3b: one factor, with the same residual covariances as Model 1	$\chi^2(32) = 106.021$, CFI = .951, TLI = .931, RMSEA = .116, SRMR = .051; vs. Model 1: $\Delta\chi^2(1) = 53.757$, $p < .001$
Model 4: four correlated factors (EE, RA, DEV, DI)	$\chi^2(29) = 35.777$, CFI = .995, TLI = .993, RMSEA = .037 [.000, .073], SRMR = .023
Latent r (burnout, DI), focused Model 1, 95% CI	.851 [.792, .911]
Latent r (burnout, DI), measurement model of the SEM	.852
r fixed at 1.0 vs. free	$\Delta\chi^2(1) = 53.757$, $p < .001$
r fixed at .90 vs. free	$\Delta\chi^2(1) = 2.957$, $p = .086$
Fornell–Larcker	√AVE burnout = .828, √AVE DI = .807; r = .852 exceeds both (criterion not met)
Composite reliability (structural measurement model)	Loadings only: BPNS .885, burnout .929, DI .881; including parcel error covariances: BPNS .784, burnout .833, DI .881
HTMT: burnout (15 items)–DI, bootstrap 95% CI	.836 [.749, .898] (without sport devaluation: .770)
Latent r with DI (parcel model), 95% CI	EE .670 [.573, .767]; RA .778 [.701, .856]; DEV .836 [.776, .896]
Latent r with DI (item model), 95% CI	EE .680 [.584, .775]; RA .732 [.645, .818]; DEV .841 [.782, .900]
HTMT with DI (items)	EE .688; RA .788; DEV .867

Table S4 (continued). Exploratory factor analysis of sport-devaluation, amotivation, and dropout-intention items (ML, oblimin).

Item	ML1	ML2	ML3
Q51	.76	−.09	.15
Q52	.91	−.04	.02
Q53	.95	.05	−.09
Q54	.65	.09	.05
Q55	.71	.11	.06
Q17	−.02	.83	.02
Q18	.02	.81	.08
Q19	.02	.96	−.09
Q20	.01	.75	.14
Q56	−.04	.03	.96
Q57	.15	.07	.65
Q58	.15	.01	.61
Q59	.33	.10	.37

Note. Factor correlations: F1–F2 = .67, F1–F3 = .76, F2–F3 = .61. Q51–Q55 = sport devaluation; Q17–Q20 = amotivation; Q56–Q59 = dropout intention.

Table S5. Standardized structural estimates in selected sensitivity models (95% CI).

Path	Main (ML)	MLR	Burnout w/o devaluation	Covariates
H1 BPNS → SDM	.690 [.601, .778]	.690 [.568, .812]	.690 [.602, .778]	.687 [.596, .777]
H2 SDM → Burnout	−.315 [−.493, −.136]	−.315 [−.523, −.107]	−.294 [−.480, −.108]	−.290 [−.476, −.105]
H4 BPNS → Burnout	−.393 [−.577, −.210]	−.393 [−.678, −.109]	−.373 [−.564, −.182]	−.410 [−.609, −.211]
H3 Burnout → DI	.794 [.674, .914]	.794 [.669, .919]	.663 [.527, .800]	.771 [.641, .901]
H5a BPNS → DI	−.050 [−.214, .114]	−.050 [−.244, .143]	−.116 [−.291, .060]	−.044 [−.218, .131]
H5b SDM → DI	−.047 [−.196, .103]	−.047 [−.186, .093]	−.100 [−.261, .061]	−.050 [−.201, .101]
H6 sequential indirect	−.173 [−.276, −.069]	−.173 [−.301, −.044]	−.135 [−.226, −.043]	−.154 [−.257, −.050]
BPNS → Burnout → DI	−.313 [−.468, −.157]	−.313 [−.550, −.075]	−.247 [−.385, −.109]	−.316 [−.484, −.149]

Note. Delta-method CIs (MLR: robust). Covariate model: n = 171. Covariate associations (β [95% CI]): Age (per 10 years) → Motivation .046 [−.081, .172]; Female → Motivation .021 [−.094, .135]; Playing experience (years) → Motivation −.124 [−.273, .025]; National-team experience → Motivation .075 [−.068, .219]; Injury (past year) → Motivation .001 [−.115, .117]; Age (per 10 years) → Burnout −.096 [−.230, .039]; Female → Burnout .018 [−.108, .144]; Playing experience (years) → Burnout .119 [−.045, .283]; National-team experience → Burnout −.022 [−.179, .135]; Injury (past year) → Burnout .042 [−.087, .170]; Age (per 10 years) → Dropout intention −.067 [−.175, .041]; Female → Dropout intention −.001 [−.101, .100]; Playing experience (years) → Dropout intention .074 [−.060, .208]; National-team experience → Dropout intention .064 [−.062, .191]; Injury (past year) → Dropout intention .061 [−.042, .164].

Table S5 (continued). Standardized estimates with the three burnout dimensions as separate correlated intervening variables.

Path (burnout dimensions as separate intervening variables)	β	95% CI	p
BPNS → SDM	.690	[.601, .778]	<.001
SDM → EE	−.244	[−.435, −.054]	.012
BPNS → EE	−.350	[−.545, −.154]	<.001
SDM → RA	−.296	[−.483, −.109]	.002
BPNS → RA	−.351	[−.544, −.158]	<.001
SDM → DEV	−.310	[−.487, −.133]	<.001
BPNS → DEV	−.354	[−.536, −.171]	<.001
EE → DI	−.024	[−.249, .201]	.834
RA → DI	.235	[−.084, .554]	.149
DEV → DI	.570	[.342, .798]	<.001
SDM → DI	−.059	[−.202, .084]	.419
BPNS → DI	−.085	[−.239, .070]	.284

Note. Fit: $\chi^2(102) = 212.799$, CFI = .958, TLI = .943, RMSEA = .079, SRMR = .047. EE = exhaustion; RA = reduced accomplishment; DEV = sport devaluation. Without DEV, RA → DI β = .643 [.374, .912], EE → DI β = .005 [−.256, .266].

Table S6. Motivation decomposition: autonomous (AM), controlled (CM), and amotivation (AMO) factors.

Path	β	95% CI	p
BPNS → AM	.638	[.535, .742]	<.001
BPNS → CM	−.018	[−.186, .150]	.835
BPNS → AMO	−.489	[−.620, −.358]	<.001
AM → BO	.095	[−.060, .250]	.230
CM → BO	−.004	[−.142, .133]	.949
AMO → BO	.561	[.411, .710]	<.001
BPNS → BO	−.413	[−.585, −.242]	<.001
BO → DI	.715	[.538, .892]	<.001
BPNS → DI	−.051	[−.231, .130]	.583
AM → DI	.009	[−.132, .149]	.902
CM → DI	.009	[−.115, .132]	.890
AMO → DI	.151	[−.032, .334]	.105
seq (a*b*d)	−.196	[−.284, −.108]	<.001
ind_am (a1*b1*d)	.043	[−.029, .116]	.244
ind_cm (a2*b2*d)	.000	[−.002, .002]	.952

Note. Fit: $\chi^2(303) = 630.448$, CFI = .923, TLI = .911, RMSEA = .079, SRMR = .066. AM indicated by intrinsic, identified, and integrated subscale means; CM by Q13–Q16; AMO by Q17–Q20. seq = sequential indirect association via amotivation and burnout; BO = athlete burnout; DI = dropout intention.

Table S6 (continued). Correlations among observed subscale means.

	Intrinsic	Identified	Integrated	Controlled	Amotivation	Exhaustion	Reduced accomp.	Devaluation	Dropout int.
Intrinsic	1	.90	.80	.07	−.34	−.23	−.32	−.30	−.31
Identified	.90	1	.82	.09	−.34	−.25	−.32	−.31	−.33
Integrated	.80	.82	1	.15	−.32	−.33	−.37	−.33	−.32
Controlled	.07	.09	.15	1	.40	.31	.14	.23	.24
Amotivation	−.34	−.34	−.32	.40	1	.56	.58	.66	.64
Exhaustion	−.23	−.25	−.33	.31	.56	1	.77	.69	.61
Reduced accomp.	−.32	−.32	−.37	.14	.58	.77	1	.77	.70
Devaluation	−.30	−.31	−.33	.23	.66	.69	.77	1	.78
Dropout int.	−.31	−.33	−.32	.24	.64	.61	.70	.78	1

Note. Pearson correlations among observed subscale means.

Table S7. Statistical power.

Analysis	Result
RMSEA-based power, test of close fit (H0 ε = .05, Ha ε = .08), df = 108, N = 173	.93
RMSEA-based power, test of not-close fit (H0 ε = .05, Ha ε = .01)	.81
Monte Carlo power (1000 converged replications of N = 173, population = estimated model): H1 / H2 / H4 / H3	1.00 / .91 / .98 / 1.00
Monte Carlo power: H5a / H5b	.11 / .10
Monte Carlo power: H6 sequential indirect association (delta-method test)	.90
